# Novel maternal autoantibodies in autism spectrum disorder: Implications for screening and diagnosis

**DOI:** 10.3389/fnins.2023.1067833

**Published:** 2023-02-02

**Authors:** Rut Mazón-Cabrera, Jori Liesenborgs, Bert Brône, Patrick Vandormael, Veerle Somers

**Affiliations:** ^1^Department of Immunology and Infection, Biomedical Research Institute, UHasselt, Transnational University Limburg, Diepenbeek, Belgium; ^2^Expertise Centre for Digital Media, UHasselt, Transnational University Limburg, Diepenbeek, Belgium; ^3^Department of Neurosciences, Biomedical Research Institute, UHasselt, Transnational University Limburg, Diepenbeek, Belgium

**Keywords:** autoantibodies, autism spectrum disorder, risk factor, pregnancy, biomarker

## Abstract

**Introduction:**

Autism spectrum disorder (ASD) is a complex neurodevelopmental disorder for which early recognition is a major challenge. Autoantibodies against fetal brain antigens have been found in the blood of mothers of children with ASD (m-ASD) and can be transferred to the fetus where they can impact neurodevelopment by binding to fetal brain proteins. This study aims to identify novel maternal autoantibodies reactive against human fetal brain antigens, and explore their use as biomarkers for ASD screening and diagnosis.

**Methods:**

A custom-made human fetal brain cDNA phage display library was constructed, and screened for antibody reactivity in m-ASD samples from the Simons Simplex Collection (SSC) of the Simons Foundation Autism Research Initiative (SFARI). Antibody reactivity against 6 identified antigens was determined in plasma samples of 238 m-ASD and 90 mothers with typically developing children (m-TD).

**Results:**

We identified antibodies to 6 novel University Hasselt (UH)-ASD antigens, including three novel m-ASD autoantigens, i.e., ribosomal protein L23 (RPL23), glyceraldehyde-3-phosphate dehydrogenase (GAPDH) and calmodulin-regulated spectrin-associated protein 3 (CAMSAP3). Antibody reactivity against a panel of four of these targets was found in 16% of m-ASD samples, compared to 4% in m-TD samples (*p* = 0.0049).

**Discussion:**

Maternal antibodies against 4 UH-ASD antigens could therefore provide a novel tool to support the diagnosis of ASD in a subset of individuals.

## 1. Introduction

Autism spectrum disorder (ASD) is a developmental disorder defined by pervasive and sustained impairments in social communication, and restricted, repetitive patterns of behavior, interest or activities (American Psychiatric Association, 2013). The prevalence of ASD is estimated at 1 in 54 children aged 8 years in the United States, 1 in 100 in Europe, and occurs four times more in males than in females (Delobel-Ayoub et al., 2020; Maenner et al., 2020).

Early recognition and diagnosis of children with ASD is essential to allow the start of an early intervention, which has been shown to lead to better outcomes (Zwaigenbaum et al., 2015). A combination of general developmental surveillance and ASD-specific early screening tests are used in primary care to identify children at risk for ASD (Hyman et al., 2020; Lipkin et al., 2020). The Modified Checklist for Autism in Toddlers (M-CHAT) and the Social Communication Questionnaire (SCQ) are some of the most used screening tests (Robins et al., 2001; Eaves et al., 2006), and are used in children between 18 and 42 months in low and high-risk populations (Baird et al., 2000). However, these tools are not designed for diagnostic purposes, and children who are identified by surveillance or screening require additional specialized evaluation for ASD diagnosis. The diagnosis of ASD is generally complex and requires a multidisciplinary approach using behavioral observations, based on the diagnostic criteria from the fifth edition of the Diagnostic and Statistical Manual of Mental Disorders (DSM-V). Multiple questionnaires have been created to support the diagnostic process, the most used are the Autism Diagnostic Observational Schedule (ADOS) and the Autism Diagnostic Interview-Revised (ADI-R), which score different behavioral patterns, such as eye contact, attention, or playing engagement (Lord et al., 1994, 2000; American Psychiatric Association, 2013).

Each of these diagnostic instruments still has limited sensitivity and specificity, and therefore only performs well, correctly identifying true positives and true negatives, in a clinical situation where the ASD prevalence is already high (Randall et al., 2018). This still leaves much room for improvement, especially when trying to identify children with ASD as early as possible. This can be achieved by improving screening, monitoring of high-risk populations, such as siblings of children with ASD, but also by the use of biomarkers which objectively measure biological characteristics of disease. Many types of ASD biomarkers have been described, ranging from structural parameters quantified using magnetic resonance imaging, physiological measurements such as electroencephalography, behavioral parameters such as eye tracking, but also a large diversity of molecular parameters, including metabolic, immune and genetic biomarkers (reviewed in Frye et al., 2019). Next to assisting diagnosis, screening, and presymptomatic detection, ASD biomarkers are potential tools to stratify ASD patients, and to provide understanding of the underlying disease mechanism at the molecular level.

The etiology of ASD is complex and multifactorial in most individuals, compounding the influence of both genetic and environmental risk factors (reviewed by de la Torre-Ubieta et al., 2016; Modabbernia et al., 2017). In the last decade, the role of immune system dysfunction in the development of ASD has received increasing attention. This link between immune dysregulation and ASD even occurs during embryonic development, where different components of the maternal immune system have been described to impact prenatal brain development, leading to an increased risk of ASD in the child. This is the case in so-called maternal immune activation, where infections or increased inflammation during pregnancy have been shown to increase the risk for development of ASD in the child (Han et al., 2021). Here, components of the maternal innate immune system mediate a more general, non-antigen-specific immune response, mainly by pro-inflammatory cytokines such as IL-6 and IL-17A (Smith et al., 2007; Choi et al., 2016). On the other hand, maternal, antigen-specific immune responses, in the form of maternal autoantibodies targeting specific fetal brain proteins, have also been linked to the development of ASD in the child (m-ASD) (reviewed by Mazon-Cabrera et al., 2019). During pregnancy, these m-ASD autoantibodies are able to pass the placenta via transplacental transport (Kim et al., 2009; Palmeira et al., 2012), and to cross the fetal blood-brain barrier, which appears to be semi-permeable during this period (Kowal et al., 2015). Recent studies revealed lactate dehydrogenase A and B (LDH-A/B), Y-box-binding protein 1 (YBX1), cytosolic PSD-95 interactor (Cypin), collapsin response mediator protein 1 and 2 (CRMP1/2), stress-induced phosphoprotein 1 (STIP1), folate receptor alpha (FOLR1), and contactin-associated protein-like 2 (CASPR2) as the main antigens targeted by m-ASD autoantibodies (Braunschweig et al., 2013; Ramaekers et al., 2013; Brimberg et al., 2016). Using animal models, the active contribution of several of these m-ASD autoantibodies in the development of autistic features in the offspring has been demonstrated. Female mice which have been immunized with a mixture of immunogenic peptides from LDH-A/B, STIP1, and CRMP1 (Jones et al., 2020), or with the extracellular portion of CASPR2 (Bagnall-Moreau et al., 2020), and which subsequently form autoantibodies against these proteins during pregnancy, produce offspring with structural brain abnormalities and autism-related behavioral changes.

A better understanding of the different brain antigens that can be targeted by maternal autoantibodies, would allow to investigate the role of these target antigens in normal brain development, and how autoantibodies directed against them might impact the development of ASD. In addition, such antibodies might prove to be valuable early biomarkers for ASD. Therefore, in this study, an unbiased screening was performed to identify novel immunoglobulin G (IgG) m-ASD antibodies. To this end, a custom cDNA phage display library was created, which represents the diversity of antigens expressed in the human fetal brain during mid gestation, and used to screen for antibody reactivity in plasma samples of mothers who have a child with ASD (m-ASD). Next, antibody reactivity against selected antigens was determined in a large collection of m-ASD and control samples, and the possible added value in supporting ASD screening and diagnosis was studied.

## 2. Materials and methods

### 2.1. Research subjects

Plasma samples from 268 mothers with a single child with ASD (m-ASD, Table 1) were obtained from the Simons Simplex Collection (SSC) of the Simons Foundation Autism Research Initiative (SFARI) in the United States (Fischbach and Lord, 2010). The children were diagnosed with ASD by their treating clinical psychologist using the DSM-IV diagnostic criteria. The maternal blood sample was obtained at ASD diagnosis of the child, at which time the child was between 3 and 7 years, and had no younger siblings. All children with ASD were white, of non-Hispanic descent, 223 (83%) were male. Relevant demographic and ASD-related clinical characteristics were obtained via SFARI Base (Fischbach and Lord, 2010), and are shown in Table 1.

**TABLE 1:** Demographic and clinical characteristics of m-ASD samples from SSC used for SAS screening and validation.

Demographic/clinical characteristics	m-ASD SAS pool (*n* = 30)	m-ASD validation (*n* = 238)
**Characteristics child diagnosed with ASD**		
Gender (male), n (%)	24 (80.0)	199 (83.6)
Age at diagnosis (years), median (IQR)	4.3 (0.3)	5.5 (1.4)
IQ < 70 ^1^, n (%)	10 (33.3)	83 (34.9)
Verbal IQ < 70 ^2^, n (%)	12 (40.0)	81 (34.0)
Regression ^3^, n (%)	18 (60.0)	98 (41.2)
Other developmental disorders ^4^, n (%)	1 (3.3)	5 (2.1)
CSS score ^5^, median (IQR)	7.5 (3.0)	8 (3.0)
CSS score ≥ 6, n (%)	28 (93.3)	224 (94.1)
CSS score ≥ 8, n (%)	15 (50.0)	124 (52.1)
ABC score > 54 ^6^, n (%)	10 (33.3)	96 (40.3)
Febrile seizures ^7^, n (%)	1 (3.3)	7 (2.9)
Non-febrile seizures ^7^, n (%)	0 (0.0)	23 (9.7)
**Characteristics mother**		
Autoimmune disorder ^8^, n (%)	10 (33.3)	66 (27.7)*
Age at delivery (years), average (SD)	32.9 (5.1)	33.0 (4.8)
Gestational age (weeks), median (IQR)	40 (1.8)	39 (2.0)

All clinical data have been collected by SFARI for the SSC.

^1^The IQ score provides an estimation of the individual cognitive ability, IQ < 70 was considered as intellectual disability.

^2^Verbal IQ provides an estimate of the individual’s verbal ability, verbal IQ < 70 was considered as language impairment.

^3^Based on ADI-R loss insert or the ADI-R loss questions.

^4^ASD individuals with other developmental disorders, including non-verbal disorders, learning disorder, and written expression disorder.

^5^ADOS Calibrated Severity Score (CSS) from Gotham et al. (2009). Values between 6 and 10 are considered moderate to severe.

^6^Total score across all items of the Aberrant Behavior Checklist, scores over 54 were considered ASD-like behavior.

^7^Combination of the ADI-R and the medical history form.

^8^History of autoimmune disorders including asthma, bowel disorders, diabetes, Hashimoto’s thyroiditis, hyper- and hypothyroidism, multiple sclerosis, psoriasis, rheumatoid arthritis, and systemic lupus erythematosus. *Missing data for 1 sample; ABC, Aberrant Behavior Checklist; ADI-R, Autism Diagnostic Interview-Revised; ADOS, Autism Diagnostic Observational Schedule; ASD, autism spectrum disorder; CSS, Calibrated Severity Score; IQ, intelligence quotient; IQR, interquartile range; m-ASD, mothers with a child with autism; SAS, Serological Antigen Selection; SD, standard deviation; SFARI, Simons Foundation Autism Research Initiative; SSC, Simons Simplex Collection.

In addition, control plasma samples from 90 mothers with typically developing children (m-TD) were collected in collaboration with University Biobank Limburg (UBiLim, Belgium) (Linsen et al., 2019). Demographic and clinical characteristics were obtained via a questionnaire. At the time of sampling, the women included in the study had a child between 3 and 7 years of age, resulting from their last pregnancy. The children of the m-TD mothers were white, of East- or West-European descent, and 44 (49%) were male. None of the m-TD mothers, or their children, presented a diagnosis of ASD, hyperactivity or learning disabilities.

A pool of 30 m-ASD plasma samples was used to screen for novel antibodies using serological antigen selection (SAS) (Table 1). A pool of plasma samples from 20-mTD, of which the youngest child was age- and gender-matched [mean (SD) age 4.3 (1.3) years, 16 (80%) were male] were used in the counterselection of the SAS procedure.

This study was conducted in accordance with the Helsinki Declaration. All m-ASD and m-TD study subjects provided written informed consent, and this study was approved by the local ethics committees of Jessa Hospital and Hasselt University.

### 2.2. Identification of novel fetal brain antigens via serological antigen selection (SAS)

Commercially obtained poly A^+^ RNA isolated from the brains of 59 spontaneously aborted male and female Caucasian fetuses, of 20−33 weeks of gestation (Clontech/Takara, Saint-Germain-en-Laye, France) was used to construct a human fetal brain cDNA phage display library according to the previously described procedure (Vandormael et al., 2017). This phage display library, expressing human fetal brain antigens, but also non-physiological peptides, was used to screen for antibody reactivity in 30 pooled m-ASD plasma samples (Table 1) using SAS, using the previously described procedure, with minor modifications (Somers et al., 2005, 2009; Quaden et al., 2020). In brief, plasma antibodies from the m-ASD SAS pool (*n* = 30) with reactivity against phage or bacterial proteins were first removed by pre-absorbing plasma to cyanogen-bromide sepharose beads (Merck, United States) coupled with phage and bacterial protein extracts. Next, in a first positive selection round of the SAS procedure, phage particles from the human fetal brain cDNA phage display library were pre-incubated with the m-ASD SAS pool in phosphate buffered saline (PBS) pH 7.4 with 2% (w/v) skimmed milk powder (MPBS), and incubated for 2 h rotating at room temperature (RT). A MaxiSorp Immuno tube (Thermo Fisher Scientific) was coated with 10 μg/ml of polyclonal rabbit anti-human immunoglobulin (Ig) G antibody (DAKO, United States) overnight at 4°C. After washing twice with PBS with 0.1% Tween20 (0.1% PBS-T) and twice with PBS, the tube was blocked with 2% MPBS for 2 h at RT, and washed again with PBS-T and PBS. The positive pre-incubation mixture was transferred to the coated Immuno tube, incubated 30 mins rotating, and 2 h while standing at RT, and washed 10 times with PBS-T and 10 times with PBS. The bound phage-antibody complexes were eluted with 100mM triethylamine, neutralized with 1M Tris-HCl pH7.4, and amplified by infecting TG1 bacteria and plating on 2xTY plates with 100 μg/ml ampicillin and 2% glucose (2xTYAG). The output of this first SAS round was used as input for three additional positive selection SAS rounds. After elution and neutralization of the output of the fourth and final positive selection round, phage particles were precipitated and used immediately (no amplification) for a round of counterselection using a pool of 20 m-TD plasma samples. Here, phage particles were pre-incubated with the m-TD plasma pool, and phage particles not bound by a rabbit anti-human IgG-coated Immuno tube were amplified by infecting TG1 bacteria and plating on 2xTYAG plates. After this final SAS round, colonies were randomly picked and stored in liquid 2xTYAG medium with 10% glycerol at −80°C. The DNA sequence of the M13 geneVI-cDNA fusion was characterized by Sanger sequencing, and the amino acid sequence of the corresponding displayed antigen was determined using DNAnalyzer software (Quaden et al., 2020). Amino acid homology between the antigen sequences and human proteins was determined using the RefSeq Select proteins database on NCBI and the blastp algorithm, sorted by *E*-value.

### 2.3. Phage enzyme-linked immunosorbent assays (phage ELISA)

Antibody reactivity in pooled or individual plasma samples against antigens displayed on the surface of purified phage clones was measured using phage ELISA, as described previously (Quaden et al., 2020). In brief, half area 96 well Microlon high-binding microplates (Greiner, Belgium) were coated overnight at 4°C with 2 μg/ml anti-M13 mouse monoclonal antibody (clone MM05T, Sino Biological, China) diluted in coating buffer (0.1 M sodium carbonate bicarbonate buffer, pH9.6). After washing [3 times using phosphate buffered saline (PBS) pH7.4 with 0.1% Tween-20, 1 time using PBS, shaking at RT], plates were blocked in PBS with 2% (w/v) skimmed milk powder (MPBS) for 2 h, shaking at 37°C. After washing, diluted antigen-expressing phage particles (7.0 × 10^11^ cfu/ml in 2% MPBS) were added, and incubated for 1 h standing at 37°C, followed by 30 mins shaking at RT. After washing, plates were incubated with diluted serum samples (1/100 in 2% MPBS) for 1 h standing at 37°C, and 30 mins shaking at RT. After washing, plates were incubated with cross-adsorbed goat anti-human IgG-Fc, conjugated with horseradish peroxidase (Bethyl, United States) diluted 1/10,000 in 2% MPBS, for 1 h shaking at RT. Finally, after washing, plates were colored with 3,3’,5,5’-tetramethylbenzidine for 10 mins in the dark, stopped using 1.8 M H_2_SO_4_, and absorbance was read at 450nm.

For each sample, the antibody reactivity is expressed as the ratio of the average optical density (OD) signal of the antigen-expressing phage clone, over the average OD of an empty phage, not expressing any antigen. All samples were tested in duplicate, and experiments were performed independently at least twice, with a coefficient of variation of the ratio < 20% between experiments. For each antigen, a cut-off for positivity was calculated as the mean antibody reactivity plus 5 times standard deviation of the non-reactive m-TD and m-ASD samples. This non-reactive sample group was determined using change-point analysis using the R package “change-point” selecting the Pruned Exact Linear Time (PELT) algorithm (Killick et al., 2012; Lardeux et al., 2016). Antibody reactivity against a panel of antigens included the combined antibody-positivity for at least one of the antigens included in the panel.

During competition ELISA, plasma samples were pre-incubated with increasing concentrations of synthetic peptide (0−30 μg/ml, >85% purity, GL Biochem, China) corresponding to the peptide sequences of UH-ASD.1, UH-ASD.8, or UH-ASD.12, or using recombinant His-tagged human RPL23 (0−30 μg/ml, Proteintech, United Kingdom) for UH-ASD.5, before being added to the respective antigen-expressing phage and to empty phage in a regular phage ELISA, as described in Quaden et al. (2020). Results are expressed as the ratio of antigen-expressing phage OD over empty phage OD. For UH-ASD.1, the antigenic peptide sequence following the cloning adaptor was used (UH-ASD.1: GKIRQPIGLF), while UH-ASD.8 was tested as three partially overlapping peptides, UH-ASD.8.1 (ASVPEYTGPRLYKEPSAKSNKFIIHNALS), UH-ASD.8.2 (SNKFIIHNALSHCCLAGKVNEPQKNRIL), and UH-ASD.8.3 (VNEPQKNRILEEIEKSKANHFLILFRDS). UH-ASD.12 was elongated with 5 N-terminal amino acids originating from the translated cloning adaptor (UH-ASD.12.1 SRPRDAATTF).

### 2.4. Post-test probability calculation

Post-test probability was calculated from pre-test odds and the positive likelihood ratio (LR+) of the applied test. Pre-test probability for full siblings was derived by multiplying the ASD prevalence of the general population (1.85%) (Maenner et al., 2020) with the relative risk for ASD in full siblings (9.3) (Hansen et al., 2019). Pre-test probability for preterm births was obtained from ASD prevalence in preterm infants (Agrawal et al., 2018). The LR+ for the M-CHAT was obtained from the pooled sensitivity (83%) and specificity (53%) of 13 studies (Yuen et al., 2018). The LR+ for the (early) SCQ at 36 months with cut-off 15, cut-off 11 for ASD with phrase speech, and cut-off 11 without phrase speech, was obtained from the sensitivity (respectively 20%, 34%, and 69%) and specificity (respectively 99%, 89%, and 89%) of a large sample of the Norwegian Mother and Child Cohort Study (Suren et al., 2019). The LR+ for the ADOS was obtained from the pooled sensitivity (94%) and specificity (80%) of 12 studies (Randall et al., 2018).

### 2.5. Statistical analysis

All statistical analyses were performed using SAS JMP Pro version 14, and a *P* < 0.05 was considered statistically significant. The presence of antibodies against individual or panels of antigens, was compared using Fisher’s exact test between m-ASD and m-TD groups.

To analyze correlations between anti-UH-ASD seropositivity in m-ASD samples, and corresponding clinical and demographic characteristics in mothers and their children with ASD, the Fisher’s exact test was used for categorical variables, the Student’s *t*-test was used for variables with parametric distribution, and the Wilcoxon/Kruskal Wallis tests was used for variables with non-parametric distribution.

## 3. Results

### 3.1. Screening for novel m-ASD antibodies reactive against human fetal brain antigens

To identify novel IgG isotype m-ASD autoantibodies with reactivity toward human fetal brain antigens, an *in vitro* representation of the proteins expressed in the developing human fetal brain was created. To this end, the mRNA expressed in human fetal brains from weeks 20 till 33 of gestation was converted to cDNA, and randomly fused to the filamentous phage gene VI, resulting in a cDNA phage display library consisting of 3.95 × 10^6^ recombinant clones. Sequencing analysis showed that the cDNA coding sequences of approximately 14%, or 5.5 × 10^5^ clones of this phage display library were fused in frame with phage gene VI, which therefore correctly express (fragments of) known human proteins. The remaining 86% of this library consists of out-of-frame cDNA fusions or fusion to non-coding cDNA sequences, which result in the expression of non-physiological peptides.

This novel fetal brain cDNA phage display library was used to screen for antibody reactivity in pooled m-ASD plasma samples from the Simons Simplex Collection (SSC) of the Simons Foundation Autism Research Initiative (SFARI) (Table 1) using SAS (Somers et al., 2005; Vandormael et al., 2017). These m-ASD samples had been obtained at ASD diagnosis of the child, at which time the child was between 3 and 7 years, and had no younger siblings. Sequencing analysis of a selection of isolated phage clones identified 149 novel candidate antigenic targets of m-ASD antibodies. Antibody reactivity against each of these individual antigens was measured by phage ELISA using pools of m-ASD (5 pools of 10 samples each) and m-TD controls (2 pools of 10 samples each), resulting in the selection of 33 antigens (called UH-ASD.1 till UH-ASD.33, Supplementary Table 1) with increased antibody reactivity in m-ASD pools (results not shown). A preliminary screening in a subpopulation of individual samples resulted in the selection of 6 antigens that were further analyzed (results not shown).

### 3.2. Identity of the fetal brain antigens targeted by m-ASD antibodies

DNA sequencing of the fusion between filamentous phage gene VI and the human fetal brain cDNA insert, allowed to determine the amino acid (aa) sequence of the antigens expressed on the phage surface (Table 2). The 6 selected antigens, called UH-ASD.1, UH-ASD.5, UH-ASD.7, UH-ASD.8, UH-ASD.12, and UH-ASD.19, were composed of protein sequences between 5 and 134 aa in length (Table 2). Of these, UH-ASD.1, UH-ASD.12, and UH-ASD.19 are non-physiological peptides resulting from the fusion to, and translation of non-coding DNA sequences. However, these antigens show partial homology with several human proteins (Table 2). On the other hand, UH-ASD.5, UH-ASD.7 and UH-ASD.8 correctly express fragments of known human proteins. The sequence of the UH-ASD.5 antigen corresponds almost entirely to the full-length sequence of the 60S ribosomal protein L23 (RPL23) of 140 aa, lacking only the 6 N-terminal aa. The UH-ASD.7 antigen consists of the last 12 C-terminal aa of the 335 aa glycolytic enzyme glyceraldehyde-3-phosphate dehydrogenase (GAPDH). Finally, UH-ASD.8 contains an internal 64 aa part (aa 1129-1192) of calmodulin-regulated spectrin-associated protein family member 3 (CAMSAP3). This sequence comprises 57 aa of the so-called CKK domain, a C-terminal domain common to CAMSAP family proteins, which is required for binding to microtubules (Baines et al., 2009).

**TABLE 2:** Sequence, origin and homology of 6 novel UH-ASD antigens.

Name UH-ASD antigen	Displayed aa sequence^1^	Aa size^2^	Identity cDNA insert (NCBI accession nr)	Type of insert^3^	Fusion in frame^4^	Homology aa level (Uniprot accession nr)^5^
UH-ASD.1	(G)KIRQPIGLF	10	(99%) Chromosome 6p24.1-25.3 (AL022098.1)	Genomic, non-coding region	N/A	- 7/8 (88%) Ankyrin repeat and SOCS box protein 7 (Q9H672) - 7/9 (78%) G protein-coupled receptor kinase 6 (P43250) - 6/8 (75%) phospholipid-transporting ATPase ABCA3 (Q99758)
UH-ASD.5	(G)GGSSGAKFR ISLGLPVGAV INCADNTGAK NLYIISVKGIK GRLNRLPAAGVG DMVMATVKK GKPELRKKVH PAVVIRQRK SYRRKDGV FLYFEDNAGVI VNNKGEMKGS AITGPVAKECA DLWPRI ASNAGSIA	134	(100%) Ribosomal protein L23 (RPL23) (NM_000978.3)	mRNA, coding region	Yes	134/134 (100%) 60S ribosomal protein L23 (P62829)
UH-ASD.7	(G)VVDLMAHMA SKE	13	(100%) Glyceraldehyde-3-phosphate dehydrogenase (GAPDH) (NM_001289746.1)	mRNA, coding region	Yes	12/12 (100%) Glyceraldehyde 3-phosphate dehydrogenase (P04406)
UH-ASD.8	(A)SVPEYTGPRLY KEPSAKSNKFIIH NALSHCCLAGKV NEPQKNRILEEIE KSKANHFLIL**FRDS** *RVPRPLIN*^6^	72	(100%) Calmodulin regulated spectrin associated protein family member 3 (CAMSAP3) (NM_001163749.1)	mRNA, coding region	Yes	64/64 (100%) Calmodulin-regulated spectrin-associated protein 3 (Q9P1Y5)
UH-ASD.12	(A)ATTF	5	(96%) Chromosome X (AL683813.10)	Genomic, non-coding region	N/A	- 5/5 (100%) Mucin-4 (Q99102) - 5/5 (100%) Teashirt homolog 3 (Q63HK5) - 5/5 (100%) Nuclear pore complex protein Nup93 (Q8N1F7.2)
UH-ASD.19	(G)LVSMTHPGEE GS[Q]FL[Q]LASG ENGTEKQEGRR RSEKNFCF	40	(100%) Chromosome 12, Homo sapiens ankyrin repeat and sterile alpha motif domain containing 1B (ANKS1B) (NG_029860.2)	Genomic, non-coding region/intron	N/A	- 12/19 (63%) chondroitin sulfate proteoglycan 5 (O95196) - 9/12 (75%) DBIRD complex subunit ZNF326 (Q5BKZ1) - 15/27 (56%) glutamate receptor ionotropic, NMDA 2D (O15399)

^1^Sequence of the antigen as expressed on the phage surface, with the first aa between parentheses representing the transition between the cloning adaptor and the cDNA insert. [Q] represents amber stop codon, which is translated into glutamine by the bacterial strain used to produce phage particles. ^2^Size of the antigen is expressed as the number of aa. ^3^Indicates the origin of the cDNA insert and the region in the RNA/DNA where the cDNA was fused to M13 gene VI. ^4^Type of fusion of the cDNA coding region with M13 gene VI. “Yes” indicates the cDNA coding region is in frame with M13 gene VI, resulting in the expression of (part of) a human protein. “N/A” indicates the cDNA fusion occurred in a non-coding region. ^5^Human proteins with homologous sequence to antigen sequence, amount and percentage of identical amino acids indicated. Top 3 hits using RefSeq Select proteins database on NCBI using the blastp algorithm, sorted by *E*-value. ^6^CAMSAP3 coding region contains an internal *Xho*I restriction site (indicated in bold), therefore the aa after this site (indicated in italic) originate from the translated pSPVI cloning vector. ASD, autism spectrum disorder; aa, amino acids; cDNA, complementary DNA; mRNA, messenger RNA; N/A, not applicable; NCBI, National Center for Biotechnology Information; nr, number; UH, University Hasselt.

### 3.3. Maternal-ASD autoantibodies to 6 novel human fetal brain antigens

Antibody reactivity against each of the 6 novel UH-ASD antigens was determined in individual plasma samples of 238 m-ASD and 90 m-TD using phage ELISA. Antibody reactivity against these individual antigens was present in 2.5% (6/238) to 19.3% (46/238) of m-ASD, and in 0% (0/90) to 14.4% (13/90) of m-TD samples (Table 3). The UH-ASD.7 (GAPDH) antigen showed the highest antibody reactivity in both the m-ASD and the m-TD groups. For each of the 6 UH-ASD antigens, antibody reactivity was higher in the m-ASD than in the m-TD group, albeit not significant. In order to be able to discriminate m-ASD from m-TD samples, the 4 antigens with the highest positive likelihood ratio (LR+), UH-ASD.1, UH-ASD.5 (RPL23), UH-ASD.8 (CAMSAP3), and UH-ASD.12, were combined into a panel. Combined antibody reactivity against at least one of the 4 UH-ASD antigens of this panel was significantly higher in m-ASD (16%) compared to m-TD (4.4%) samples (*p* = 0.005). Similarly, a second panel of antigenic targets was composed of the 3 autoantigens UH-ASD.5 (RPL23), UH-ASD.7 (GAPDH), and UH-ASD.8 (CAMSAP3), which showed more autoantibody reactivity in m-ASD samples (25.2%) compared to m-TD controls (15.6%), but could not significantly discriminate between these two groups (*p* = 0.075).

**TABLE 3:** Antibody reactivity to individual and combinations of UH-ASD antigens in m-ASD and m-TD samples.

	Autoantigen identity	m-ASD n (%)^1^	m-TD n (%)^2^	LR+	*p*-value
**Individual UH-ASD antigens**					
UH-ASD.1	N/A	18/238 (7.6%)	2/90 (2.2%)	3.40	0.118
UH-ASD.5	RPL23	4/238 (1.7%)	0/90 (0%)	N/A	0.578
UH-ASD.7	GAPDH	46/238 (19.3%)	12/90 (13.3%)	1.45	0.256
UH-ASD.8	CAMSAP3	13/238 (5.5%)	2/90 (2.2%)	2.46	0.373
UH-ASD.12	N/A	6/238 (2.5%)	1/90 (1.1%)	2.27	0.678
UH-ASD.19	N/A	39/238 (16.4%)	13/90 (14.4%)	1.13	0.737
**Panels of UH-ASD antigens ^3^**					
UH-ASD.1/5/8/12		38/238 (16.0%)	4/90 (4.4%)	3.59	0.005**
UH-ASD.5/7/8	All autoantigens	60/238 (25.2%)	14/90 (15.6%)	1.62	0.075

^1^Number and percentage of anti-UH-ASD positive m-ASD samples. ^2^ Number and percentage of anti-UH-ASD positive m-TD samples. ^3^ Combined antibody reactivity against at least one of the antigens in the panel. **p ≤ 0.01. ASD, autism spectrum disorder; CAMSAP3, calmodulin regulated spectrin associated protein family member 3; GAPDH, glyceraldehyde-3-phosphate dehydrogenase; LR+, positive likelihood ratio; m-ASD, mothers with a child with autism; m-TD, mothers with typically developing children; RPL23, ribosomal protein L23; UH, University Hasselt.

### 3.4. Specificity of maternal antibodies to UH-ASD antigens

**FIGURE 1:**
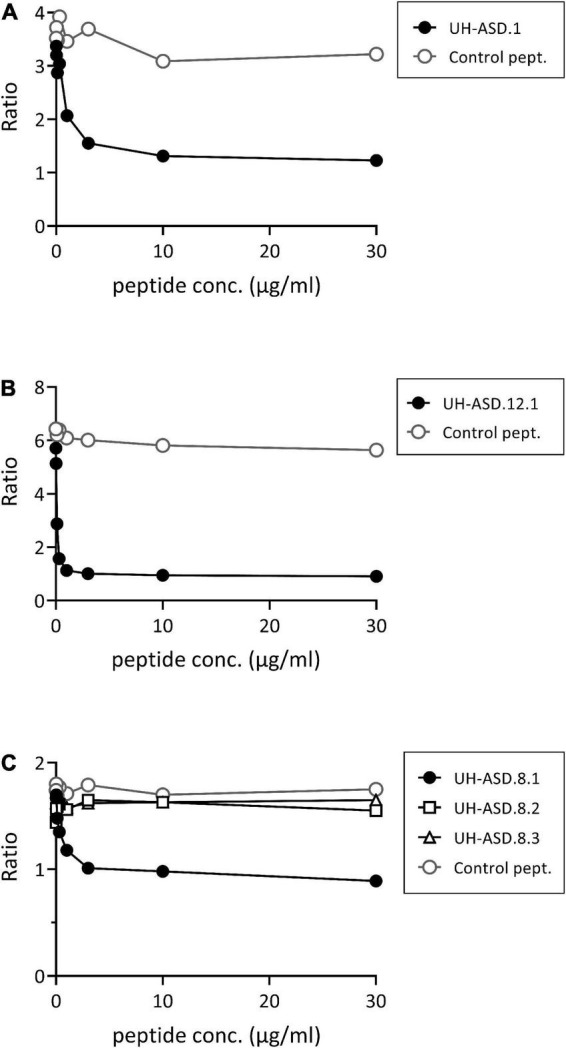
Competition for binding phage-displayed antigens using synthetic peptides. In a phage ELISA, an antibody-positive plasma sample was pre-incubated in solution with increasing amounts of synthetic peptide, corresponding to the respective displayed antigens of UH-ASD.1 **(A)**, UH-ASD.12.1 **(B)**, or three partially overlapping peptides for UH-ASD.8 (8.1, 8.2, and 8.3) **(C)**. As a control the samples were pre-incubated with similar amounts of a control peptide. Antibody reactivity is expressed as the ratio of OD specific phage/OD empty phage. ASD, autism spectrum disorder; UH, University Hasselt.

Determination of antibody reactivity against our UH-ASD antigens has been performed using phage ELISA, where each respective antigen was displayed at the surface of phage particles. In order to confirm that the m-ASD antibodies specifically bind to the displayed antigen, a competition ELISA using synthetic peptide or recombinant protein was performed for the antigens of the panel composed of UH-ASD.1, UH-ASD.5 (RLP23), UH-ASD.8 (CAMSAP3), and UH-ASD.12. Clear competition could be seen using synthetic peptides for the short UH-ASD.1 and UH-ASD.12 antigens, which were formed by the expression of non-coding sequences (Figures 1A, B). Antibody specificity to the 1129 to 1192 aa fragment of CAMSAP3, expressed by UH-ASD.8, was tested using three overlapping synthetic peptides (UH-ASD.8.1, 8.2, and 8.3). Since the UH-ASD.8.1 peptide showed evident competition, while the partially overlapping peptide UH-ASD.8.2 did not, antibody reactivity could be narrowed down to a region between aa 1129 and 1146 of CAMSAP3 (Figure 1C). On the other hand, competition using recombinant full length RPL23 protein could not lower binding to UH-ASD.5 (RLP23) under the tested conditions (data not shown).

### 3.5. Correlation between anti-UH-ASD antibodies and clinical parameters

Demographic and clinical parameters of mothers, and of their child with ASD, were compared in m-ASD samples that were seropositive or seronegative for antibodies against the panel of UH-ASD.1/5/8/12 antigens (Table 4). The maternal parameters that were investigated, i.e., maternal age at birth and maternal autoimmune disease, were not significantly different in m-ASD samples that were antibody positive or negative for this antigenic panel. Likewise, presence or absence of maternal antibody reactivity against the UH-ASD.1/5/8/12 antigens, was not correlated with any of the parameters of the child with ASD, including gender, gestational age, age at diagnosis, Calibrated Severity Score (CSS), Aberrant Behavior Checklist (ABC), IQ and Verbal IQ, regression, presence of other developmental disorders, and febrile and non-febrile seizures.

**TABLE 4:** Correlation between clinical characteristics and m-ASD antibody reactivity.

Characteristic	UH-ASD seropositive^1^ (*n* = 38)	UH-ASD seronegative^2^ (*n* = 200)	*p*-value
Gender (male), n (%)	31 (81.6)	168 (84.0)	0.811
IQ < 70 ^3^, n (%)	15 (39.5)	68 (34.0)	0.579
Verbal IQ < 70 ^4^, n (%)	13 (34.2)	68 (34.0)	1.000
Regression ^5^, n (%)	19 (50.0)	79 (39.5)	0.281
Other developmental disorders ^6^, n (%)	2 (5.3)	3 (1.5)	0.181
CSS score ^7^, median (IQR)	8 (3.0)	8 (3.0)	0.401
CSS score > 6, n (%)	36 (94.7)	188 (94.0)	1.000
CSS score > 8, n (%)	22 (57.9)	102 (51.0)	0.482
ABC score > 54 ^8^, n (%)	19 (50.0)	123 (61.5)	0.209
Febrile seizures ^9^, n (%)	1 (2.6)	6 (3.0)	1.000
Non- febrile seizures ^9^, n (%)	4 (10.5)	19 (9.5)	0.770
Autoimmune disorder in the mother ^10^, n (%)	10 (27.0)*	56 (28.0)	1.000
Maternal age at birth (years), average (SD)	34.0 (5.0)	32.8 (4.7)	0.178
Gestational age (weeks), median (IQR)	39 (2.0)	39 (2.0)	0.830

^1^m-ASD samples with antibody reactivity against at least one of the UH-ASD.1/5/8/12 antigens.

^2^m-ASD samples without antibody reactivity against the UH-ASD.1/5/8/12 antigens.

^3^The IQ score provides an estimation of the individual cognitive ability, IQ < 70 was considered as intellectual disability.

^4^Verbal IQ provides an estimate of the individual’s verbal ability, verbal IQ < 70 was considered as language impairment.

^5^Based on ADI-R loss insert or the ADI-R loss questions.

^6^ASD individuals with other developmental disorders, including non-verbal disorders, learning disorder, and written expression disorder.

^7^ADOS Calibrated Severity Score (CSS) from Gotham et al. (2009). Values between 6 and 10 are considered moderate to severe.

^8^Total score across all items of the Aberrant Behavior Checklist, scores over 54 were considered ASD-like behavior.

^9^Combination of the ADI-R and the medical history form.

^10^History of autoimmune disorders including asthma, bowel disorders, diabetes, Hashimoto’s thyroiditis, hyper- and hypothyroidism, multiple sclerosis, psoriasis, rheumatoid arthritis and systemic lupus erythematosus. *Missing data for 1 sample; ABC, Aberrant Behavior Checklist; ADI-R, Autism Diagnostic Interview-Revised; ADOS, Autism Diagnostic Observational Schedule; ASD, autism spectrum disorder; CSS, Calibrated Severity Score; IQ, intelligence quotient; IQR, interquartile range; m-ASD, mothers with a child with autism; SAS, Serological Antigen Selection; SFARI, Simons Foundation Autism Research Initiative; SSC, Simons Simplex Collection; UH, University Hasselt.

### 3.6. Added value of anti-UH-ASD antibody reactivity in ASD diagnosis

**FIGURE 2:**
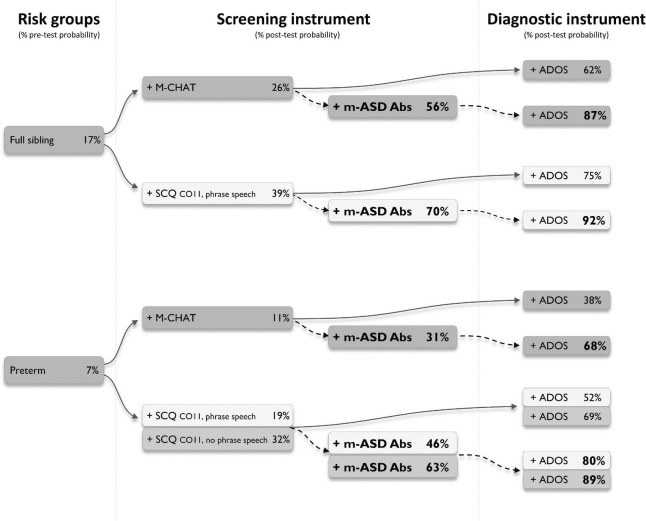
Simulated diagnostic trajectories with and without additional m-ASD antibody testing. Different diagnostic trajectories were studied, and the stepwise increase in post-test probability for having autism spectrum disorder (ASD) was calculated, when a person would be part of an ASD risk group (full ASD sibling or preterm), would be positive for one of the indicated screening instruments (M-CHAT or SCQ), and would be positive for the ADOS as a diagnostic instrument (solid arrows). For each trajectory, the added value of testing for anti-UH-ASD.1/5/8/12 antibodies was calculated (dashed arrows). Pre-test probability for full siblings was obtained from Killick et al. (2012), Maenner et al. (2020), for preterm births from Lardeux et al. (2016). Positive likelihood ratios to calculate post-test probabilities for M-CHAT were obtained from Hansen et al. (2019), for early SCQ from Agrawal et al. (2018), and for ADOS from Randall et al. (2018). ADOS, Autism Diagnostic Observational Schedule; CO, cut-off; M-CHAT, Modified Checklist for Autism in Toddlers; m-ASD Abs, maternal autism spectrum disorder antibodies; SCQ, Social Communication Questionnaire.

Finally, we explored whether testing for antibodies against the UH-ASD.1/5/8/12 antigens could provide added value during the diagnostic process. We selected a number of diagnostic trajectories involving three mains steps, starting from at-risk groups, followed by the application of a screening instrument, and finally a diagnostic instrument (Figure 2). In these trajectories, the effect of additional testing for anti-UH-ASD.1/5/8/12 antibodies during the screening step, was calculated. In each consecutive step, the pre- or post-test probability for actually having ASD was calculated in case the tests gave a positive result (Figure 2). To this end, we used reported prevalences and positive likelihood ratios for siblings of a child with ASD (Hansen et al., 2019; Maenner et al., 2020), or children with preterm birth (Agrawal et al., 2018) as risk groups, for the M-CHAT (Yuen et al., 2018), or the early SCQ (Yuen et al., 2018; Suren et al., 2019) as screening instruments, and finally, for the ADOS (Randall et al., 2018) as a diagnostic instrument. When testing for anti-UH-ASD.1/5/8/12 antibodies would be added in combination with the M-CHAT screening, the post-test probability after a positive ADOS could be increased from 62 to 87% for siblings, and from 38 to 68% for preterm births (Figure 2). Screening siblings and children with preterm birth using the regular SCQ cut-off of 15, already gave sufficiently high post-test probabilities after ADOS, of approximately 90% or higher, even in the absence of additional antibody testing (not shown). On the other hand, the lower SCQ cut-off of 11 is frequently used to increase the sensitivity in younger children (Barnard-Brak et al., 2016; Moody et al., 2017), but because of lower specificity, this also decreases the probability for correct diagnosis after ADOS (Figure 2). Using this lower SCQ cut-off, additional testing for anti-UH-ASD.1/5/8/12 antibodies again increased the post-test probability after a positive ADOS, from 75 to 92% for siblings, and from 52 to 80% for preterm births, in case phrase speech was present in the SCQ. In the absence of phrase speech during the SCQ, testing for our antibody panel would only cause a meaningful increase in post-test probability for the preterm births, increasing from 69 to 89%.

## 4. Discussion

In the current study, we have identified novel m-ASD antibodies against 6 antigens originating from a human fetal brain library, and validated their reactivity in mothers of a child with autism and mothers of typically developing children. Three of these antigens, UH-ASD.5, UH-ASD.7, and UH-ASD.8, correspond to parts of proteins expressed in the human fetal brain, respectively RPL23, GAPDH, and CAMSAP3. This is the first time that these three proteins have been identified as targets of maternal autoantibodies in ASD. Furthermore, antibody reactivity against a panel of 4 UH-ASD antigens could increase the probability of a correct early diagnosis in certain risk populations, when applied in conjunction with early screening instruments.

We have used the SAS technology to screen for novel m-ASD autoantibodies. A custom-made cDNA phage display library was generated from mRNA expressed in human fetal brains, which thus forms an *in vitro* representation of the antigens expressed during human fetal brain development. The use of the SAS technology has proven to be a successful method for identifying novel antibody biomarkers in other diseases such as rheumatoid arthritis, axial spondylarthritis, multiple sclerosis and spinal cord injury (Somers et al., 2008, 2011; Palmers et al., 2016; Quaden et al., 2020). This is the first time that human fetal brain antigens have been used in a comprehensive screening for targets of m-ASD antibodies, as previous studies have screened starting from protein extracts of Rhesus macaque fetal brain (Braunschweig et al., 2013), or used a candidate-based approach (Brimberg et al., 2016). With approximately 5.5 × 10^5^ protein coding genes cloned in the correct reading frame, our phage display library has an average 29-fold coverage of the number of protein coding genes in the human genome (Ezkurdia et al., 2014). However, because of the starting material used to create this library, it will be highly enriched for genes which are expressed in the human brain during weeks 20 to 33 of fetal development. During this period, several processes of brain development have been linked to ASD, such as neurogenesis, neuronal migration, synaptogenesis and gliogenesis, and the beginning of myelination (reviewed by Estes and McAllister, 2016).

Our SAS screening resulted in the identification of 149 novel candidate fetal brain antigens. Initial testing for antibody reactivity in m-ASD and m-TD samples resulted in six antigens, UH-ASD.1, UH-ASD.5 (RLP23), UH-ASD.7 (GAPDH), UH-ASD.8 (CAMSAP3), UH-ASD.12, and UH-ASD.19 with increased antibody reactivity in m-ASD samples. Further validation in a large number of m-ASD and m-TD plasma samples showed that antibody reactivity against at least one of the 4 antigens with the highest LR+, UH-ASD.1, UH-ASD.5, UH-ASD.8, and UH-ASD.12, is significantly more present in m-ASD than in m-TD, with a sensitivity of 16.0% and a specificity of 95.6%. Presence of these antibodies did not correlate with the clinical characteristics that were investigated, neither of the mothers, nor from their children with ASD. Consequently, these antibodies could not identify subpopulations of ASD individuals based on phenotype, probably due to their limited presence. Of note, mothers that were positive for these antibodies did not show increased prevalence of a number of autoimmune diseases that are characterized by the presence of autoantibodies, such as rheumatoid arthritis and systemic lupus erythematosus. Therefore, the antibodies with self-reactivity identified in this study do not seem to originate from intrinsic autoimmune processes or disorders in the mother, although maternal autoimmunity has been implicated with increased risk for development of autism in the child (Chen et al., 2016; Wojcik et al., 2017; Spann et al., 2019).

Early identification of children with ASD is critical for early intervention, which has been shown to increase the chance of a better outcome and reduce the long-term healthcare cost (Peters-Scheffer et al., 2012; Estes et al., 2015; Landa, 2018). In recent years, general developmental screening, but also screening using ASD-specific tools, such as M-CHAT and SCQ, have been introduced in primary care routines as a first-line tool for identifying children at risk (Robins et al., 2001; Eaves et al., 2006; Hyman et al., 2020). However, the performance of these screening tools is not always sufficient to identify ASD individuals, especially in young children. When applied in a universal setting, these screening instruments have the limitation of low probability of correctly identifying children with ASD, because of the relatively low ASD prevalence in the general population (Yuen et al., 2018; Suren et al., 2019). Even focusing on the at-risk populations of siblings, and children with preterm births, leaves much room for improvement, depending on the screening instrument used. In this study, we determined the added value of our newly identified antibody biomarkers in calculating the post-test probability for correct diagnosis in a number of different scenarios, combining at-risk populations, the M-CHAT and early SCQ screening tools, and the ADOS for diagnosis. Adding antibody testing to the M-CHAT screening instrument greatly increased the confidence in the final diagnosis. Testing positive for anti-UH-ASD.1/5/8/12 antibodies could also clearly increase the post-test probability when combined with screening using SCQ obtained at 36 months, especially when the lower cut-off value of 11 was used, which allows a much higher sensitivity in younger children (Barnard-Brak et al., 2016; Moody et al., 2017). In children who show early phrase speech, which represent about 76% of the children with ASD (Suren et al., 2019), this benefit of additional antibody testing showed the largest added value. A major advantage of these antibody biomarkers is that they can be easily measured in the maternal blood at early time points during the child’s development. One of the limitations is that the m-ASD sample would only be positive in about 16% of cases using these specific biomarkers. Application of a panel of additional early biomarkers, which can be other similar maternal serological markers, but also EEG, eye tracking, genetic or metabolic markers, could increase the number of children with ASD that are correctly recognized in an early phase (Vanaken et al., 2021).

Functional studies in animal models have shown that maternal autoantibodies which target human fetal brain proteins can enter fetal brain tissue *in utero*, induce modifications in the developing brain, and trigger ASD-like behaviors in the offspring (Garty et al., 1994; Zimmerman et al., 2007; Croen et al., 2008; Martin et al., 2008; Singer et al., 2008, 2009; Goines et al., 2011; Palmeira et al., 2012; Braunschweig et al., 2013; Brimberg et al., 2013; Jones et al., 2020). Studying such autoantibodies and their target antigens can therefore provide a better understanding of the underlying causes of disease. In the future, such antibodies might be used to better inform patients, they might allow physicians to make targeted therapy choices, and can also provide possible novel points of entry for pharmaceutical companies, to tackle the underlying disease process at the molecular level. In this study, we found 3 novel m-ASD autoantibodies, directed respectively against RPL23, the C-terminus of GAPDH, and part of the tubulin-binding domain (CKK domain) of CAMSAP3. RPL23 is a structural protein of the large ribosomal subunit (60S), and has not yet been associated with ASD. However, many components of the translation machinery have been described as autism risk genes (reviewed in Chen et al., 2019), and a strong signature or translational downregulation has been described in male fetal brains after maternal immune activation (Kalish et al., 2021). The dehydrogenase GAPDH is a glycolytic enzyme which catalyzes the oxidation of glyceraldehyde-3-phosphate into 1,3-bisphosphoglycerate. Anti-GAPDH autoantibodies are not specific for m-ASD, and have been described in autoimmune diseases such as systemic lupus erythematosus (SLE), and have been related to cognitive dysfunction in these patients (Delunardo et al., 2016). Anti-GAPDH antibodies have also been detected in patients with schizophrenia and major depression, and administration of these immunoglobulins to mice leads to cognitive and behavioral alterations (Delunardo et al., 2016). Remarkably, autoantibodies against other glycolytic enzymes, i.e., lactate dehydrogenase (LDH) A and B, and neuron-specific enolase, have been described in m-ASD samples (Braunschweig et al., 2013; Ramirez-Celis et al., 2020). These autoantibodies might even actively contribute to the development of autistic behaviors, as female mice which have been immunized against 4 antigens, including LDH-A and LDH-B, produce offspring with altered development and social interactions (Jones et al., 2020). Finally, CAMSAP3 is an essential protein in the organization of non-centrosomal microtubules, stabilizing their minus ends through the CKK domain (Baines et al., 2009). Through its regulation of these microtubules, CAMSAP3 is a key player in cell polarity in specific cell types such as neurons and epithelial cells (Toya et al., 2016; Pongrakhananon et al., 2018; Chen et al., 2020). CAMSAP3 is a leading player in axon development, and its loss in neurons leads to formation of multiple axons (Pongrakhananon et al., 2018). Alterations in the morphology of neurons, changes in polarity, and consequently, modifications in the connectivity, have been described both in individuals with ASD and in models of ASD, which makes CAMSAP3 an interesting candidate target in the study of the etiology of the disease (Donovan and Basson, 2017). Of note, RPL23, GAPDH, and CAMSAP3 are intracellular proteins, and whether or how autoantibodies targeting them would affect brain development, would need to be further explored. It has been shown that autoantibodies targeting the intracellular proteins LDH-A and LDH-B, STIP1 and CRMP1 are able to induce autism-related behavioral changes in a mouse model, however, but how these antibodies target their antigens is also still unknown (Jones et al., 2020). In addition, RPL23, GAPDH, and CAMSAP3, and most of the described m-ASD antigens described by others, are not uniquely expressed in the fetal brain, and are also found postnatally, and in other tissues.

For the remaining three anti-UH-ASD antibodies, the exact *in vivo* antigens are currently not known, as they are reactive against peptides generated from the translation of non-coding sequences (UH-ASD.1, UH-ASD.12, and UH-ASD.19). These peptides probably form mimotopes, which are sequences that mimic the epitopes the antibodies were initially formed against. Nevertheless, some of these mimotope antigens show aa homology with proteins implicated in ASD, such as Teashirt homolog 3, whose gene deletion causes a syndrome with high autism prevalence in affected patients (Caubit et al., 2016), and ASD-like behavioral deficits in a knockout mouse model (Chabbert et al., 2019). Still, whether anti-UH-ASD antibodies are actually directed against these, or other human proteins, remains to be investigated.

## 5. Limitations

The results of this study should be interpreted while considering some limitations. Our novel fetal brain cDNA phage display library probably cannot account for autism-specific (neo)antigens that would arise from specific mutations or altered gene splicing, or for genes specifically expressed before, or after 20 to 33 weeks of gestation. Moreover, because of our cloning method, expression of human proteins is biased toward C-terminal fragments. In addition, bacterial expression of our cDNA phage display library gives constraints with respect to protein folding, post-translational modification and the expression of membrane proteins.

Secondly, although phage particles of the UH-ASD.5 antigen express 134 of the 140 amino acids of human RPL23, competition experiments could not definitely confirm that anti-UH-ASD.5 antibody reactivity was directed against the RPL23 protein. This might be caused by differences in folding between RPL23, expressed on the phage surface and recombinant RPL23, or anti-UH-ASD.5 antibody reactivity might be directed against an epitope formed by the fusion site between the adaptor used for cloning, and the beginning of the RPL23 sequence. Therefore, as our described reactivity values of the antibody panel rely upon UH-ASD.5, further determination of the exact epitope is required before use in clinical settings.

Finally, as we used maternal plasma samples taken at the time of diagnosis of the child, we need to assume that the presence of antibodies at the time of sampling is representative for the situation during the pregnancy, 3−7 years earlier. In most persons, the presence and levels of protective antibodies against pathogens, or of disease-associated autoantibodies, are stable over a period of several years to decades (Amanna et al., 2007; Gkrouzman et al., 2021). However, the antibody reactivities described in the current study should be confirmed in maternal plasma samples obtained during pregnancies of children which went on to develop autism later, such as from the Finnish or Norwegian birth cohorts (Lampi et al., 2011; Magnus et al., 2016), which are coupled to clinical data involving long-term follow up of child development. Finally, in the future, the specificity of our UH-ASD antibodies should be studied in maternal samples coupled to children who do not have ASD, but show conditions that are often ASD co-morbidities such as ADHD, epilepsy or mental impairment.

## 6. Conclusion

Maternal antibodies against a combination of 4 novel human fetal brain antigens are increased in our cohort of mothers of children with autism. These antibodies can provide a novel tool to support the early recognition of children with ASD, providing added value when used in combination with early screening tools in at-risk populations. Moreover, the discovery of autoantibodies directed against the fetal brain antigens RPL23, GAPDH, and CAMSAP3, could provide further understanding the role of maternal autoantibodies in ASD pathophysiology.

## Data availability statement

The datasets presented in this article are not readily available because data are available upon reasonable request. The authors commit to making the relevant anonymized reactivity and patient data available for a specified purpose approved by the institution and the principal investigator of the study, and with a signed data access agreement. Requests to access the datasets should be directed to VS, veerle.somers@uhasselt.be.

## Ethics statement

The studies involving human participants were reviewed and approved by the Medical Ethics Committee UHasselt. The patients/participants provided their written informed consent to participate in this study.

## Author contributions

PV, BB, and VS designed the study. RM-C and PV acquired the experimental data and drafted the manuscript. RM-C, JL, and PV performed the data analysis. BB, VS, and JL revised the manuscript critically for the important intellectual content. All authors were involved in interpretation of data and approved the final draft for publication.
