# Supplementary material for: Novel maternal autoantibodies in autism spectrum disorder: Implications for screening and diagnosis
Source: Front Neurosci. 2023 Feb 2;17:1067833. doi: 10.3389/fnins.2023.1067833 (PMC9932693; doi:10.3389/fnins.2023.1067833)
Supplement: Supplementary file 1 [file Table_1.docx]

| **Name**  **UH-ASD antigen** | **Displayed aa**  **sequence ^1^** | **Aa size ^2^** | **Identity cDNA insert (NCBI accession nr)** | **Type of insert ^3^** | **Fusion**  **in frame ^4^** |
| --- | --- | --- | --- | --- | --- |
| UH-ASD.1 | (G)KIRQPIGLF | 10 | (99%) Chromosome 6p24.1-25.3 (AL022098.1) | Genomic, non-coding region | N/A |
| UH-ASD.2 | (V)MAHMASKE | 9 | (100.0%) Glyceraldehyde-3-phosphate dehydrogenase (GAPDH) (NM_001289746.1) | mRNA, coding/3'UTR region | Yes |
| UH-ASD.3 | (W)DY | 3 | (100.0%) STIP1 homology and U-box containing protein 1 (STUB1) (NM_001293197.1) | mRNA, coding/3'UTR region | Yes |
| UH-ASD.4 | (W)*REGGHTEYQ*EEELLKDLEESVQDKNTNTLFSVKLEEELSVEGNTDINTDLKD | 53 | (71%) SATB homeobox 1 (SATB1) (NM_001322876.1) | mRNA, coding region | Yes |
| UH-ASD.5 | (G)GGSSGAKFRISLGLPVGAVINCADNTGAKNLYIISVKGIKGRLNRLPAAGVGDMVMATVKKGKPELRKKVHPAVVIRQRKSYRRKDGVFLYFEDNAGVIVNNKGEMKGSAITGPVAKECADLWPRIASNAGSIA | 134 | (100.0%) Ribosomal protein L23 (RPL23) (NM_000978.3) | mRNA, coding region | Yes |
| UH-ASD.6 | (G)GLHAAACAAAMSLVIPEKFQHILRVLNTNIDGRRKIAFAITAIKGVGRRYAHVVLRKADIDLTKRAGELTEDEVERVITIMQNPRQYKIPDWFLNRQKDVKDGKYSQVLANGLDNKLREDLERLKKIRAHRGLRHFWGLRVRGQHTKTTGRRGRTVGVSKKK | 163 | (100.0%) Ribosomal protein S18 (RPS18) (NM_022551.2) | mRNA, 5'UTR/coding region | Yes |
| UH-ASD.7 | (G)VVDLMAHMASKE | 13 | (100.0%) Glyceraldehyde-3-phosphate dehydrogenase (GAPDH) (NM_001289746.1) | mRNA, coding region | Yes |
| UH-ASD.8 | (A)SVPEYTGPRLYKEPSAKSNKFIIHNALSHCCLAGKVNEPQKNRILEEIEKSKANHFLILFRDSRVPRPLIN | 72 | (100.0%) Calmodulin regulated spectrin associated protein family member 3 (CAMSAP3) (NM_001163749.1) | mRNA, coding region | Yes |
| UH-ASD.9 | (G)EAPTEEEESEALP[Q]EENTAHLRPPAKIHRINNNSY | 36 | (100.0%) Leucine rich repeat containing 37A (LRRC37A) (NM_014834.4) | mRNA, coding/3'UTR region | Yes |
| UH-ASD.10 | (A)ALGVLESDLPSAVTLLKNLQEQVMAVTAQVKSLTQKVQAGAYPTEKVRCTQTVSIP | 57 | (100.0%) Neuroguidin (NGDN), transcript variant 2 (NM_015514.1) | mRNA, coding region | Yes |
| UH-ASD.11 | (G)DGVEDHSPLMYHISLVDLLAACAEGKNVYTEIKCTSLLPLEDVVSVVTHEDCITEVKMAYVNFVNHCYVDTEVEMKEIYTSNHIWTLFENFTLDMARVCSKREKRVADPTLEKYVLSVVLDTINAFFSSPFSENSTSLQTHQTIVVQLLQSTTRLLEGTAAAN | 164 | (100.0%) Inositol 1,4,5-trisphosphate receptor type 3 (ITPR3) (NM_002224.3) | mRNA, coding region | Yes |
| UH-ASD.12 | (A)ATTF | 5 | (96%) Chromosome X (AL683813.10) | Genomic, non-coding region | N/A |
| UH-ASD.13 | (D)NF[Q]LRAWWLGRFSPFQSETSAANMLRQIIGQAKKHPSLIPLFVFIGTGATGATLYLLRLALFNPDVCWDRNNPEPWNKLGPNDQYKFYSVNVDYSKLKKERPDF | 105 | (100.0%) NDUFA4, mitochondrial complex associated (NDUFA4) (NM_002489.3) | mRNA,5'UTR/coding region | Yes |
| UH-ASD.14 | (A)GGLRGAGGGAGAFAELAGLAMAAARPARGPELPLLGLLLLLLLGDPGRGAASRVPRPLIN | 61 | (100.0%) Smoothened, frizzled class receptor (SMO) (NM_005631.4) | mRNA, 5'UTR/coding region | Yes |
| UH-ASD.15 | (A)IGNF | 5 | (100.0%) Chromosome 12  (NC_000012.12) | Genomic, non-coding region | N/A |
| UH-ASD.16 | (A)VIPALREAEVGGSRDQEIETILADTVKPHLY | 32 | (100.0%) Ferric chelate reductase 1 like (FRRS1L) (NM_014334.3) | mRNA, 3'UTR region | N/A |
| UH-ASD.17 | (G)RLTPVIPALWEAETGGSPEVGSSRPALPTWRNPISTKNTKLPGRGGACL | 50 | (100.0%) 5'-nucleotidase, cytosolic II (NT5C2), transcript variant 1 (NM_0012229.5) | mRNA, 3'UTR region | N/A |
| UH-ASD.18 | (G)QINT | 5 | (100.0%) Ankyrin repeat and zinc finger domain containing 1 (ANKZF1) (NM_001282792.1) | mRNA, coding region | No |
| UH-ASD.19 | (G)LVSMTHPGEEGS[Q]FL[Q]LASGENGTEKQEGRRRSEKNFCF | 40 | (100.0%) Chromosome 12  (NC_000012.12) | Genomic, non-coding region | N/A |
| UH-ASD.20 | (A)PGKGLL[Q]R[Q]PPPRGWRSTLDTKPSVSETEGSWGLATPEVGAVGC | 45 | (100.0%) Acid sensing ion channel subunit 1 (ASIC1), transcript variant 3 (NM_001256830.1) | mRNA, 3'UTR region | N/A |
| UH-ASD.21 | (G)ASSEAWETAW | 11 | (100.0%) Solute carrier family 25 member 6 (SLC25A6)  (NM_001636.3) | mRNA, coding region | N/A |
| UH-ASD.22 | (G)LTPVIPALWDAEVGGSQGQQFETSLANIVKPHLY | 35 | (100%)Chromosome 2 (NC_000002.12) | Genomic, non-coding region | N/A |
| UH-ASD.23 | (G)LTPVIPALWEAETGGSRGQEIETILANTVKPRLY | 35 | (93%) Chromosome 7 (AC244506.2) | Genomic, non-coding region | N/A |
| UH-ASD.24 | (G)TGAERRSINTY | 12 | (100%) Secernin 3 (SCRN3) (NM_001412202.1) | mRNA, 5'UTR/coding region | No |
| UH-ASD.25 | (G)FVSHY | 6 | (100.0%) Phospholipase A2 group XIIA (PLA2G12A) (NM_030821.4) | mRNA,3'UTR region | N/A |
| UH-ASD.26 | (V)WNV | 4 | (100.0%) Hemoglobin subunit gamma 2 (HBG2) (NM_000184.2) | mRNA, 5'UTR/coding region | No |
| UH-ASD.27 | (G)YSQLREGCFLDTPPPGPQTARLQE | 25 | (99%)Chromosome 2 (NC_000002.12) | Genomic, non-coding region | N/A |
| UH-ASD.28 | (V)VNI[Q]HRNQYFSPGIYGGSGSVNVVESSRKRKGRKETARRPWRMWIVWISWSVDSS | 56 | (100.0%) SIN3 transcription regulator family member A (SIN3A) (NM_015477.2) | mRNA, coding region | No |
| UH-ASD.29 | (P) | 1 | (100.0%) Tubulin beta 2A class IIa (TUBB2A) (NM_001310315.1) | mRNA, coding region | N/A |
| UH-ASD.30 | (V)KVND | 5 | (100.0%) Chromosome 12  (NC_000012.12) | Genomic, non-coding region | N/A |
| UH-ASD.31 | (G)GSGDGSCDSNPCWAETGQSGADV | 24 | (100.0%) Cyclin dependent kinase 2 associated protein 2 (CDK2AP2) (NM_005851.4) | mRNA, 5'UTR region | No |
| UH-ASD.32 | (G)EDVRLPVGKDKGMRLQRELAATASAKLCLWGLLQKPGLRKIRHHWEGCVGARGPSWFSRALWKLGLGLGGIWQSWVTLAFPVNVAM | 87 | (100.0%) PITH domain containing 1 (PITHD1)(NM_020362.4) | mRNA, 3'UTR region | N/A |
| UH-ASD.33 | (G)GGGGGGEAAGAGAGAGAGGGGGGGG[Q]T | 28 | (100.0%) Transcription factor 4 (TCF4) (NM_001083962.2) | mRNA, 5'UTR region | No |

Supplementary table 1. Sequence and origin of 33 novel UH-ASD antigens

^1^Sequence of the antigen as expressed on the phage surface, with the first aa between parentheses representing the transition between the cloning adaptor and the cDNA insert. [Q] represents amber stop codon, which is translated into glutamine by the bacterial strain used to produce phage particles. ^2^Size of the antigen is expressed as the number of aa. ^3^Indicates the origin of the cDNA insert and the region in the RNA/DNA where the cDNA was fused to M13 gene VI. ^4^Type of fusion of the cDNA coding region with M13 gene VI. “Yes” indicates the cDNA coding region is in frame with M13 gene VI, resulting in the expression of (part of) a human protein. “No” indicates the cDNA coding region is not fused in frame with M13 gene VI. ”N/A” indicates the cDNA fusion occurred in a non-coding region. ASD, autism spectrum disorder; aa, amino acids; cDNA, complementary DNA; mRNA, messenger RNA; N/A, not applicable; NCBI, National Center for Biotechnology Information; nr, number; UH, University Hasselt; UTR, untranslated region.
